# Undernutrition and malaria in pregnancy – a dangerous dyad?

**DOI:** 10.1186/s12916-016-0695-2

**Published:** 2016-09-19

**Authors:** Holger W. Unger, Per Ashorn, Jordan E. Cates, Kathryn G. Dewey, Stephen J. Rogerson

**Affiliations:** 1 Department of Medicine at the Doherty Institute, The University of Melbourne, Melbourne, Victoria Australia; 2 Simpson Centre for Reproductive Health, Edinburgh Royal Infirmary, Edinburgh, UK; 3 Department of Paediatrics, University of Tampere School of Medicine, Tampere, Finland; 4 Department for International Health, University of Tampere School of Medicine, Tampere, Finland; 5 Department of Paediatrics, Tampere University Hospital, Tampere, Finland; 6 Department of Epidemiology, University of North Carolina-Chapel Hill, Chapel Hill, NC USA; 7 Program in International and Community Nutrition and Department of Nutrition, University of California, Davis, CA USA

**Keywords:** Pregnancy, Malaria, Nutrition, Low birthweight, Fetal growth restriction

## Abstract

**Background:**

In low-resource settings, malaria and macronutrient undernutrition are major health problems in pregnancy, contributing significantly to adverse pregnancy outcomes such as preterm birth and fetal growth restriction. Affected pregnancies may result in stillbirth and neonatal death, and surviving children are at risk of poor growth and infection in infancy, and of non-communicable diseases in adulthood. Populations exposed to macronutrient undernutrition frequently reside in malaria-endemic areas, and seasonal peaks of low food supply and malaria transmission tend to coincide. Despite these geographic and temporal overlaps, integrated approaches to these twin challenges are infrequent.

**Discussion:**

This opinion article examines the current evidence for malaria-macronutrition interactions and discusses possible mechanisms whereby macronutrient undernutrition and malaria may interact to worsen pregnancy outcomes. Macronutrient undernutrition dysregulates the immune response. In pregnant women, undernutrition may worsen the already increased susceptibility to malarial infection and could impair development of protective immunity to malaria, and is likely to exacerbate the impact of placental malaria on fetal growth. Malarial infection, in turn, can drive nutritional depletion; poor gestational weight gain and weight loss in pregnancy increases the risk of adverse pregnancy outcomes. Despite a commendable number of studies and trials that, in isolation, attempt to address the challenges of malaria and undernutrition in pregnancy, few dare to venture beyond the ‘single disease – single solution’ paradigm. We believe that this may be a lost opportunity: researching malaria–nutrition interactions, and designing and implementing integrated interventions to prevent and treat these commonly co-existing and intertwining conditions, may markedly reduce the high burden of preterm birth and fetal growth restriction in affected areas.

**Conclusion:**

We call for more collaboration between researchers studying malaria and nutrition in pregnancy, and propose a research agenda to address this important twin health problem.

## Background

Pregnancy and early childhood (the first 1000 days of life) are critical periods that determine short- and long-term health outcomes [1]. In low- and middle-income countries (LMICs), two important barriers to a successful pregnancy outcome are maternal undernutrition, which contributes to 800,000 neonatal deaths annually [2], and malaria, estimated to cause approximately 900,000 low birthweight (LBW) deliveries and over 100,000 infant deaths annually [3, 4]. Infant undernutrition, including fetal growth restriction (FGR), contributes to 45 % of deaths in children under 5 years, and may lead to chronic disease in adulthood [2, 5]. Ending the malaria epidemic and addressing the nutritional needs of children, adolescent girls and pregnant women form key components of the recently-launched Global Strategy for Women’s Children’s and Adolescent’s Health for 2016–2030 [6].

**Fig. 1:**
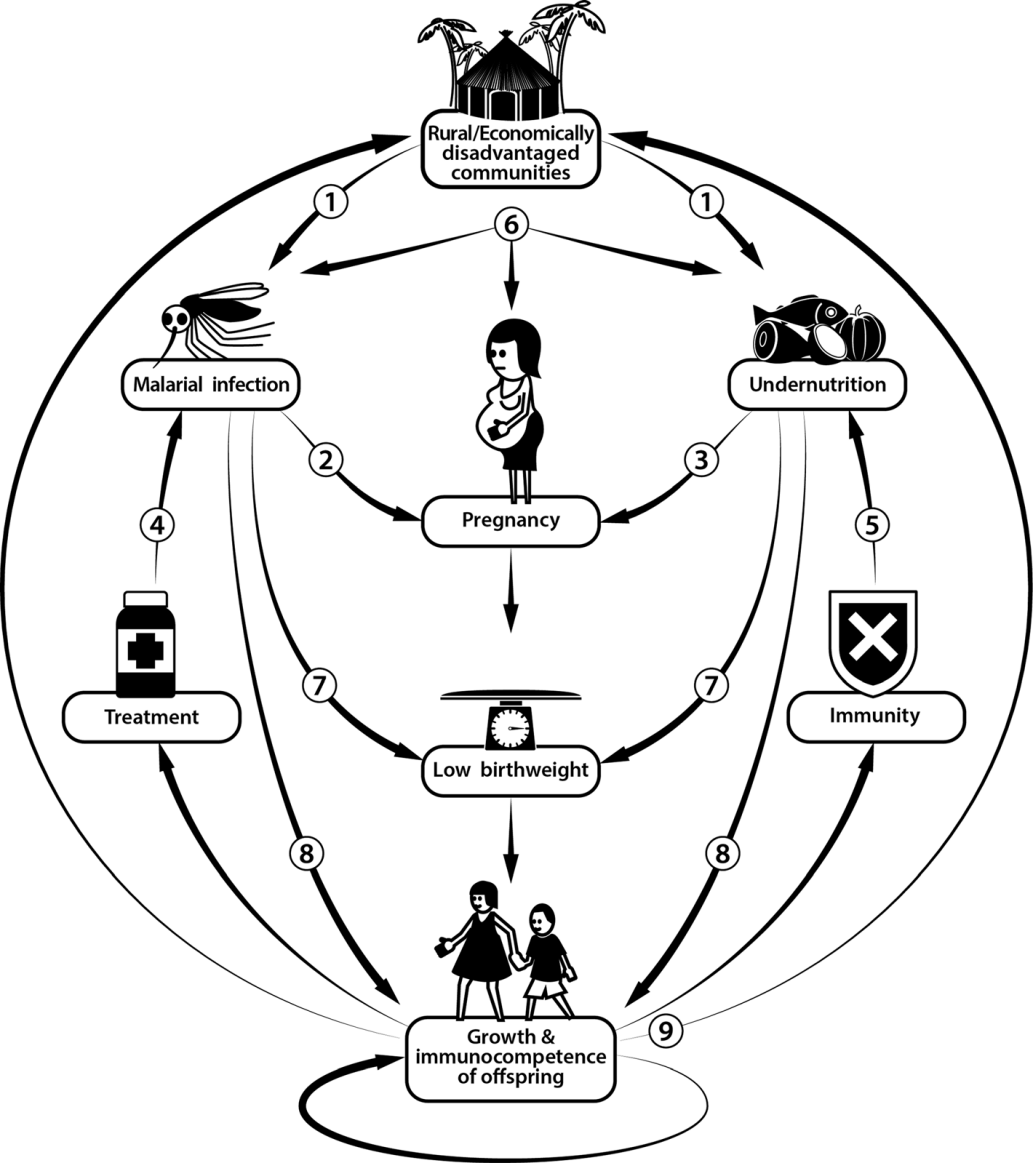
Interactions between macronutrient undernutrition and malaria. 1) Undernutrition is common in areas where malaria prevalence is high, and rural and economically disadvantaged communities are often most affected. 2) Pregnant women are more likely to be bitten by malaria-infected mosquitoes, and are more susceptible to malaria infection. 3) Undernutrition is common in pregnant women, and short inter-pregnancy intervals may lead to nutritional depletion. 4) Undernutrition may impair antimalarial treatment efficacy. 5) Nutritional status and nutrient supplementation may affect antimalarial immunity. 6) Malaria and undernutrition may interact to worsen pregnancy outcomes. 7) Both malaria and undernutrition are important causes of low birthweight. 8) Malaria and undernutrition may affect growth and immunocompetence in the offspring. 9) These combined effects of malaria and undernutrition may have long-term health and socioeconomic consequences extending into adult life and passed on transgenerationally

The World Health Organization’s (WHO) Sustainable Development Goals include ending hunger and malnutrition; reducing maternal, newborn and child mortality; and ending infections such as malaria [7]. In LMICs, populations are often affected by both hunger and malaria [2, 8], and the two may interact (Fig. 1). Nutritional status and intake of specific nutrients may affect immunity, modulating an individual’s ability to control and clear infection [9]. In turn, infection and associated inflammatory processes increase energy expenditure and protein catabolism, draining nutritional reserves. Of the many potential nutrition-infection interactions in pregnancy [10], malaria is especially important, being the leading preventable cause of LBW in Africa.

A growing body of evidence suggests that malaria and maternal undernutrition interact to worsen pregnancy outcomes. However, interventions to protect pregnant women and their fetuses from macronutrient undernutrition and gestational malaria remain poorly integrated. Our belief is that maternal nutrient deficiency remains a neglected public health problem, and that few successful interventions in this area have adequately dealt with malaria as a cofactor*.* In this call for increased collaboration between malaria and nutrition experts, we discuss the evidence for malaria–nutrition interactions in pregnancy, with a focus on macronutrient undernutrition, as this remains relatively understudied, notwithstanding the importance of micronutrient deficiencies. Macronutrient undernutrition refers to insufficient consumption of carbohydrates, fats and proteins, and is typically assessed using anthropometric measures in resource-limited settings. We summarise currently available tools to prevent and treat macronutrient undernutrition and malaria in pregnancy and outline key research questions that may advance our understanding of gestational malaria–nutrition interactions with a view to developing novel approaches to improve pregnancy outcomes in LMICs.

### Burden of malaria and macronutrient undernutrition in pregnancy

Each year, 125 million pregnant women, mostly in sub-Saharan Africa and Asia, are at risk of malaria infection [8]. Worldwide, at least 10 % of pregnant women are undernourished, defined as a pre-pregnancy body mass index (BMI) of less than 18.5 kg/m^2^, with prevalence being highest, again, in LMICs in Africa and Asia [2]. Severe maternal undernutrition is rare outside of famine and conflict situations, but moderate undernutrition is common, and associated with LBW [2, 11, 12].

### Current evidence for malaria–macronutrition links in pregnancy

There is evidence for geographical, socio-economic, temporal and mechanistic links between malaria and macronutrient undernutrition (Fig. 1). Global distribution maps of malaria transmission and undernutrition statistics clearly highlight a broad geographical overlap. Undernourished individuals, including pregnant women, are more likely to live in economic and environmental circumstances that favour malaria exposure [2]. Arguably, these overlaps in disease geography and exposure risk alone provide sufficient proof of need to design interventions that prevent and treat both malaria and undernutrition in pregnancy and infancy.

Malaria and macronutrient undernutrition in pregnancy are also linked temporally. In pregnant Gambian women, the incidence of FGR, preterm birth (PTB) and malaria were all highest late in the hunger season [13], the part of the rainy season before harvest begins. In the same setting, food supplements (high-energy groundnut biscuits) had most impact on birthweight over this period [14]. These results suggest that simple environmental co-incidence of both conditions worsens pregnancy outcomes (whether in an additive or synergistic manner remains unknown), and/or that acute macronutrient shortages increase the risk and impact of gestational malaria (effect measure modification). These findings urgently require confirmation.

Macronutrient undernutrition is associated with increased malaria morbidity and mortality in children and non-pregnant adults, suggesting important immunological interactions [15, 16]. Malaria, in turn, causes nutritional depletion and worsens child undernutrition [17]. Such interactions are likely to exist in pregnancy [18]. Whether undernutrition alters pregnant women’s risk of contracting malaria infection is unknown, but in the Democratic Republic of Congo women with low mid-upper arm circumference (MUAC) and low BMI were most likely to have high placental parasite loads [19].

Studies suggest that the effect of *Plasmodium falciparum* infection on fetal growth and birthweight is more pronounced amongst women with macronutrient undernutrition compared to well-nourished women [18, 20, 21]. In the Democratic Republic of Congo, the effects of maternal *P. falciparum* parasitaemia on uteroplacental flow and fetal growth were most pronounced amongst undernourished women (low MUAC or BMI) [18, 20]. Similarly, in Kenya, an association between peripheral *P. falciparum* infection and reduced birthweight was only reported amongst women with a low BMI [21].

### Fetal and postnatal effects

While poor nutrition and malaria have important adverse consequences for maternal health, the developing fetus is most affected. This can have severe immediate as well as long-term consequences – the foundations for an effective immune system, adequate growth, and short- and long-term health are laid in utero [5]. Macronutrient undernutrition and malarial infection have been independently associated with FGR, PTB and stillbirth [1, 11, 22], and the risk of adverse outcomes may be highest when pregnant women are affected by both [18]. To date, it remains unknown whether malaria and undernutrition act additively or even synergistically to affect pregnancy outcome, or whether effect measure modification of the impact of malaria on birthweight by nutritional status is present [18].

Malaria and macronutrient undernutrition in pregnancy have each been associated with increased infant morbidity and mortality. Many of the 900,000 LBW deliveries attributed to malaria take place in areas where maternal nutrition is poor, but the extent to which undernutrition contributes to this burden is unknown [3]. In utero exposure to malaria or undernutrition may affect the immunocompetence of the offspring, and could thus alter the risk of malaria-related morbidity and mortality in infancy [5, 23, 24]. Gestational malaria and undernutrition have been associated with suboptimal postnatal growth, suggesting in utero insults have lasting effects on the growth trajectory [25]. Growth faltering in utero and early infancy results in short adult stature, itself a risk factor for LBW, highlighting the cyclical, transgenerational effects of poor nutrition (Fig. 1) [26]. Moreover, LBW due to maternal macronutrient deficiency has been epidemiologically linked to adulthood non-communicable diseases [5, 27, 28].

### Effects of nutrition on antimalarial antibody

In undernourished children, research indicates that antibody responses to various infections remain adequate, yet most such studies involve a small number of subjects and rarely assess antibody functionality [29]. By contrast, there is evidence to suggest that undernutrition reduces immunological response to vaccines [30] and diminishes antibody-mediated antimalarial immunity [31, 32].

Limited studies have assessed the effect of maternal macronutrient undernutrition on acquisition or maintenance of *P. falciparum* antibody responses. In pregnancy, the most important antibody target is *Pf*VAR2CSA, a parasite protein inserted in the surface of infected erythrocytes that mediates placental parasite sequestration [33]. Susceptibility to malaria is greatest in first pregnancy, when women lack antibody to the *Pf*VAR2CSA protein [33], and this antibody has been correlated with protection against placental malaria, LBW and anaemia [34].

In Papua New Guinea and Malawi, maternal MUAC was not associated with levels of antibody to *Pf*VAR2CSA-expressing infected erythrocytes but maternal weight was inversely correlated with antibody levels to *P. falciparum* merozoite surface protein 2 and to *Pf*VAR2CSA [35]. In a different study, Malawian women with a BMI of less than 18.5 kg/m^2^ at first antenatal visit had higher antibody levels to *Pf*VAR2CSA at 36 gestational weeks than those with a normal or raised BMI [36]. A similar relationship was found for low socio-economic status: the association between undernutrition and antibody levels may be a result of increased exposure to malaria due to poor housing and limited access to bed nets and antimalarials [36]. In the same cohort, lipid-based nutrient supplementation (LNS) during pregnancy did not improve anti-malarial antibody responses [36]. To untangle the overlapping exposures relating to malaria immunity and undernutrition, further randomised trials of interventions may be needed.

Even if antibody levels or functionality are not affected, maternal macronutrient undernutrition might affect the ability of phagocytic cells to clear infection [37]. Undernutrition and placental malaria appear to reduce transplacental transfer of protective antibodies to some pathogens, but not to others [38, 39]. Malaria in pregnancy and undernutrition may also affect vaccine response in the offspring [29, 40] – such potential intergenerational effects require urgent evaluation.

### Effects of malaria and nutrition on cellular immune responses

Pregnancy is characterised by a switch from a pro-inflammatory T-helper 1 (Th1) to a Th2 cytokine response, which protects the fetal allograft from rejection by the maternal immune system [41]. Although inflammation plays critical roles in implantation, placentation and birth, at other time points, it may contribute to adverse pregnancy outcomes including LBW [41]. Placental malaria causes local inflammatory cytokine and chemokine production, which is associated with LBW, PTB and FGR [33], while macronutrient undernutrition has been associated with reduced T-cell activation (due to lowered circulating glucose), a decrease in total T-cell numbers and an altered cytokine profile [42]. The impact of the dysregulated cellular immune response in undernourished women on fighting malarial infection remains poorly understood. Infections other than malaria are likely to play an important role too [43], in particular if resulting in systemic and/or placental inflammation, which have been associated with adverse pregnancy outcomes [44]. These infections may be more common and detrimental amongst undernourished women.

### Effects on fetal nutrient supply

Placental malaria affects transplacental transport of nutrients, including amino acids and glucose [45]; this is most apparent when there is intervillositis (accumulation of malaria pigment-containing mononuclear cells) [33]. In undernourished women, compensatory upregulation of transplacental amino acid transport can occur in response to reduced amino acid availability [46], but this could be impaired by placental malaria, thereby limiting fetal nutrient supply even further.

### Effects on placental development

A growing body of evidence suggests that malaria infection in early pregnancy increases the risk of adverse pregnancy outcomes independently of infections following organogenesis and placentation [47]. While the impact of pre-conceptional malaria is unknown, nutritional status at conception has important consequences for embryonic and placental development and fetal growth [48], and maternal weight and gestational weight gain both influence placental weight [49]. Further, both malaria and undernutrition are known to negatively affect trophoblast migration and invasion capacity [50, 51], important determinants of the development of maternal placental blood flow. In Congolese women, early pregnancy parasitaemia was associated with increased uterine artery resistance in undernourished but not in well-nourished women [20]. Malaria and undernutrition may both affect vascular development of the fetal placental circulation [52].

### Effects of malaria and undernutrition on growth-related hormones

#### Leptin

The adipokine leptin increases Th1 cell numbers and cytokine secretion [42]. Low levels of leptin are seen in undernourished women [53] and are predictive of mortality in undernourished children [54]; they have also been reported in placental malaria [55]. Nevertheless, the consequences of suppressed maternal leptin for pregnancy outcome remain unknown.

#### Cortisol

Both protein-energy undernutrition and malaria infection are associated with elevated serum cortisol levels [56, 57], which may translate into increased fetal cortisol exposure. Undernutrition can reduce the function of 11-β hydroxysteroid dehydrogenase, the gatekeeper for fetal cortisol exposure; effects of placental malaria on its activity remain unknown [22]. Increased fetal cortisol levels have been associated with reduced thymus size and impaired lymphopoiesis and with effects on the hypothalamus–pituitary–adrenal axis and on both cellular and humoral immunity [53].

#### IGF axis

The IGF axis is a principal driver of fetal growth [22]. Inflammatory cytokines, such as TNFα and IL-1β, produced in response to malaria decrease placental amino acid uptake [45] and decrease IGF1 [22], as does undernutrition [58], suggesting malaria and undernutrition might act synergistically to affect fetal growth. In utero stress can cause neuroendocrine changes that predispose to diseases such as diabetes; altering the regulation of IGF levels may be one possible pathway [59].

### The conundrum of co-existing macro- and micronutrient deficiencies

Pregnant women with macronutrient undernutrition are prone to concurrent micronutrient deficiencies, with iron deficiency probably being the most common. Attempts should be made to take these into account when studying malaria–nutrition relationships [15, 60]. In children, iron deficiency may protect from severe malaria, and iron supplementation without antimalarial chemoprophylaxis exacerbates infection and mortality risk [61]. Iron deficiency may reduce the risk of placental malaria [62], yet it increases the risk of maternal anaemia and could cause LBW directly or via anaemia-dependent effects [63]. Iron supplementation combined with adequate malaria prevention appears not to increase the risk of maternal *Plasmodium* infection [63–65].

Furthermore, both malaria infection and macronutrient malnutrition, particularly protein deficiency, are causes of maternal anaemia, itself associated with LBW [2, 22]. Other infectious causes of iron deficiency and anaemia, such as intestinal helminth infection, may also be important, given their potential modifying impact on malaria, nutrient absorption and LBW risk [66, 67]. Folate supplementation in pregnancy prevents neural tube defects, but may increase infection risk and, in high doses, could result in failure of antifolate antimalarials [68]. Multiple micronutrient supplements decrease LBW, FGR and stillbirths [69]; however, whether deficiencies of micronutrients such as zinc, calcium and vitamin A interact with malaria in pregnancy remains unclear [70].

### Assessment of macronutrient nutritional status in pregnancy

Assessment of maternal nutrition forms part of the WHO’s focused antenatal care strategy [71], which states that women with a low booking BMI (<18.5 kg/m^2^) should receive support. Many pregnant women still seek antenatal care late, and BMI tends to increase as a result of gestational weight gain [12]; therefore, women who might benefit from nutrition–malaria interventions could be missed. MUAC varies little over gestation, and can help to identify undernourished women who are at risk of delivering a LBW baby [72], but cut-points for low MUAC and BMI may not identify the same women [72]. Maternal height less than 155 cm (indicating childhood stunting) and low gestational weight gain are also associated with PTB and FGR [26]. These measures need to be evaluated in relation to malaria risk, although the best and most applicable nutrition-related predictor of adverse pregnancy outcome remains to be clarified.

### Current tools to combat malaria and macronutrient undernutrition in pregnancy

To protect pregnant women from malaria, the WHO currently recommends the use of insecticide-treated bed nets, monthly intermittent preventive treatment with sulphadoxine-pyrimethamine from second trimester onwards, and malaria case detection and management [73]. By contrast, current management of macronutrient undernutrition is largely limited to dietary advice and improving women’s economic status, although studies demonstrate that balanced energy-protein supplements are beneficial [74, 75]. Nevertheless, given the omnipresence of poverty, dietary advice may have limited effect in LMICs [76].

Balanced energy-protein supplementation was shown to improve pregnancy outcomes and increase birthweight by an average of 100 g in undernourished women [77]. In The Gambia, daily dietary supplementation with high-energy groundnut biscuits (4.3 MJ/day) improved gestational weight gain and increased birthweight; effects were greatest in primigravidae and in the hunger (malaria) season [14]. Lipid-based nutrient supplements (LNS) may be alternative strategies to improve birthweight and gestational length. In Burkina Faso, daily LNS (1.56 MJ/372 kcal, 14.7 g protein and micronutrients) increased gestational length amongst women with a low baseline BMI and anaemia and had greatest impact during the rainy season [78]. In Ghana and Malawi, daily LNS (118 kcal, including 20 micronutrients) increased length (~4–5 mm) and weight (~50 g) at birth, and had greatest effects on birth size amongst primigravid, anaemic and short-statured women (Ghana) [79]. In Malawi, LNS was most beneficial in women who had HIV infection or malaria [80]. Multiple micronutrient and iron/folic acid supplements may also significantly increase birthweight and reduce LBW and stillbirth, but effect sizes tend to be modest [77]. Taken together these results suggest that nutritional supplementation may be most beneficial in vulnerable women, such as primigravidae and women living with HIV, who have no or impaired immunity to pregnancy-associated malaria and are at high risk of undernutrition, malaria and LBW [22]. Adolescent women are another potential target group, being at increased risk of undernutrition, malaria and adverse pregnancy outcomes [81].

Other approaches to improve maternal nutritional status whilst reducing malaria risk could be of use. Intermittent preventive treatment with sulphadoxine-pyrimethamine and azithromycin reduced the risk of LBW, improved birthweight and gestational length, and prevented growth faltering in early infancy in two of three clinical trials [82, 83]. In Papua New Guinea and Malawi, it was associated with increased gestational weight gain (Ashorn P, unpublished observations) [12]. Beyond azithromycin’s antimalarial effects, underlying mechanisms could involve reduction in maternal energy expenditure following clearance of infections, increased appetite once infection resolves, increased nutrient availability to the fetus, and effects on maternal gut microbiota that improve dietary energy harvest [84]. Infection and nutrition-related inflammatory processes have been associated with adverse pregnancy outcomes, including PTB [44]. Azithromycin has anti-inflammatory activity and may clear other infections responsible for inflammatory responses [83].

### Building the evidence base

Many pregnant women are at risk of both undernutrition and malaria, yet few studies have investigated gestational malaria–nutrition interactions. Given their potential consequences for fetal growth and development, an expanded evidence base on malaria–nutrition interactions in pregnancy is urgently needed, with closer collaboration between malariologists, nutritionists and reproductive health specialists. Table 1 summarises current research gaps.

**Table 1:** Key research gaps in the study of nutritional status and malaria in pregnancy

	Research area
Burden	Evaluate the extent to which undernutrition and malaria in pregnancy co-exist • DHSS data, pooled analysis of relevant pregnancy cohort studies
Risk of malaria	Determine the relationship between maternal nutritional status and risk of malaria parasitaemia • Longitudinal cohort studies with frequent molecular infection monitoring
Effect modification	Determine whether malaria and nutritional status interact to worsen pregnancy outcomes (LBW, SGA, PTB, stillbirth) • Pooled analysis of pregnancy cohort studies, new longitudinal studies measuring most known confounders
Conception and early pregnancy	Examine effects of undernutrition and malaria at conception and in early pregnancy on outcomes • Longitudinal observational or interventional studies beginning before conception or in first trimester including repeated measures of malaria and nutritional status • Effect of early pregnancy malaria and undernutrition on rates of miscarriage, stillbirth, LBW
Concomitant micronutrient deficiencies	Investigate possible interactions between deficiencies of micronutrients such as iron and gestational malaria • Pregnancy cohort studies that evaluate malaria indices, micronutrient status, and pregnancy outcomes
Infant effects	Study the effects of maternal undernutrition and malaria on infant morbidity, mortality, growth and infection risk
	Evaluate the consequences of maternal undernutrition and malaria for development of the infant’s immune system Investigate whether children born with LBW due to undernutrition and/or malaria would benefit from increased nutritional support and malaria prevention in infancy • Longitudinal cohort studies with well-supported follow-up mechanisms • Randomised trials of packaged malaria and nutrition interventions for at-risk infants
Anthropometrics, cytokines and hormones	Relate anthropometric measurements in pregnancy to biochemical markers of nutritional status (albumin), regulators of the nutrition/immunity interface (e.g. leptin), and adverse pregnancy outcomes in malaria-endemic areas • Determine the interplay between undernutrition, Th1/Th2 balance, placental malaria-associated inflammation, and LBW
Gut function Antimalarial efficacy	Examine effects of environmental enteric dysfunction, and of changes in gut microbiome, on maternal nutrition and malaria susceptibility Evaluate the impact of macronutrient status on efficacy of antimalarials (including IPTp) in pregnant women.
Gestational weight gain	Evaluate strategies to improve gestational weight gain in undernourished women to counteract the effect of placental malaria • Combining nutrient or energy supplementation with antimalarial interventions Investigate novel mechanisms by which IPTp may work to improve maternal weight gain • Microbiome studies, pre and post IPTp, and using different IPTp candidates • Intestinal helminth – nutrition - malaria interactions
Antenatal care	Design and test approaches that integrate malaria-nutrition interventions • Combined or factorial trials of nutritional supplementation and malaria detection and prevention • Identify target groups for enhanced nutritional support and malaria protection, e.g. undernourished women, primigravidae, and adolescents.
Immunity	Examine the relationship between nutritional status and antibody-mediated immunity to PM, controlling for differences in malaria exposure among participants
	Evaluate the effect of nutritional intervention on antibody-mediated immunity to PM
Placental studies	Investigate potential overlapping effects of malaria and macronutrient undernutrition on placental nutrient transport

*DHSS* demographic and health surveillance system, *PM* placental malaria, *LBW* low birthweight, *IPTp* intermittent preventive treatment in pregnancy

Better mapping the overlapping burdens of undernutrition and malaria infection in pregnancy could be complemented by pooled analysis of data from pregnancy cohort studies that measured both nutritional and malariometric indices to confirm effect measure modification of the malaria–birthweight relationship by nutritional status. Studies are needed in early pregnancy and periconceptionally [48], when impacts on fetal development and immunological programming may be most profound. Determining the long-term effects of malaria and undernutrition, and research that aims to untangle the effects of mixed macro- and micronutrient deficiencies for malaria immunity and placental malaria-associated adverse pregnancy outcomes, are needed. Similarly, the impact of poor nutrition in pregnancy on efficacy of malaria treatment and vaccines should be assessed.

### Potential new interventions

There is mounting evidence that antenatal identification and tailored management of high-risk mothers may prevent adverse pregnancy outcomes due to malaria and undernutrition, and further research, such as pooled analysis, may provide additional evidence for this approach. Potential target groups include adolescent, primigravid, undernourished, HIV-infected or anaemic women. These women in particular may benefit from enhanced malaria prevention strategies combined with effective nutritional supplementation, which can be delivered conjointly through antenatal care systems. Drugs that improve gestational weight gain in undernourished women, or that clear other deleterious infections (e.g. *Chlamydia trachomatis*) whilst providing antimalarial protection, also deserve further evaluation [12, 83].

## Conclusions

Macronutrient undernutrition, alone and in concert with placental malaria, can deleteriously impact fetal growth, and could affect both immunocompetence (and infection risk and mortality in the offspring), and long-term health. To meet the World Health Assembly’s targets for improving nutrition status, including decreases in maternal anaemia and LBW, improvement of malaria prevention will also be necessary given the major contributions of malaria to both LBW and anaemia.

Joint efforts by nutritionists and malaria experts are needed to determine the burden and significance of malaria-nutrition interactions in pregnancy, and to develop interventions that protect pregnant women and their children from this dangerous dyad.

## Abbreviations

BMI: body mass index
FGR: fetal growth restriction
LBW: low birthweight
LMICs: low-and-middle-income countries
LNS: lipid-based nutrient supplements
MUAC: mid-upper arm circumference
PTB: preterm birth
Th: T helper cell
WHO: World Health Organization
